# Supplementary material for: Disorder versus two transport lifetimes in a strongly correlated electron liquid
Source: Sci Rep. 2017 Sep 4;7:10312. doi: 10.1038/s41598-017-10841-w (PMC5583181; doi:10.1038/s41598-017-10841-w)
Supplement: Supplementary file 1 — Supplementary Material [file 41598_2017_10841_MOESM1_ESM.pdf]

Supplementary Material

Disorder versus two transport lifetimes in a strongly correlated electron liquid

Patrick B. Marshall, Honggyu Kim, and Susanne Stemmer

Materials Department, University of California, Santa Barbara, CA 93106-5050, USA

HAADF images of the planar defects

Figure S1 shows HAADF images of a $SmTiO_3/SrTiO_3/SmTiO_3$ quantum well structure with 2 SrO layers grown, recorded along $[001]_O$. The planar defects identified in this sample are stacking faults, where the $(010)_O$ planes are shifted by ½ *c* or ¼ *c* along the growth direction. Closer inspection of the Sm displacements, which form a "zig-zag" pattern, shows that the stacking faults in Figs. S1 (a) and (b) have ½ *c* and ¼ *c* shifts, respectively. The defects are not related to strain relaxation of the quantum well structure since the planes are shifted parallel to the out-of-plane direction. Rather, the planar defects could originate from the atomic steps at the substrate surface, which is difficult to confirm in HAADF images due to the absence of image contrast between $DyScO_3$ and $SmTiO_3$ and projection issues.

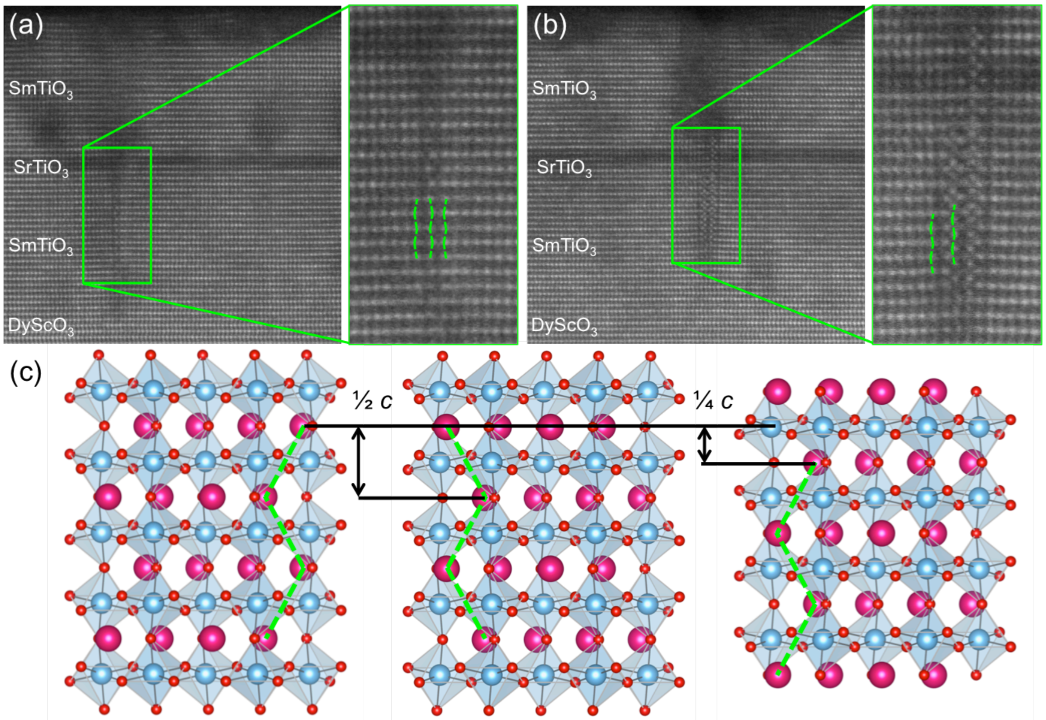

Figure S1. (a,b) HAADF images of planar defects in $SmTiO_3/SrTiO_3$(2 SrO layers)/$SmTiO_3$ quantum well structures recorded along $[100]_O$. (c) Atomic structure models are shown for stacking faults with ½ *c* (middle) and ¼ *c* (right) shifts. The pink, blue, and red circles in (c) represent Sm, Ti and O atoms, respectively. The lines are guides to the eye.

Reciprocal Space Map

Figure S2 shows a reciprocal space map of a 10 nm $SmTiO_3$/10 SrO/10 nm $SmTiO_3$ quantum well structure. The map was acquired by aligning to the 116 orthorhombic peak of the $DyScO_3$ substrate. The alignment of the in-plane lattice parameters (horizontal axis) of the $SmTiO_3$ layers and the substrate indicates that the layers are fully strained.

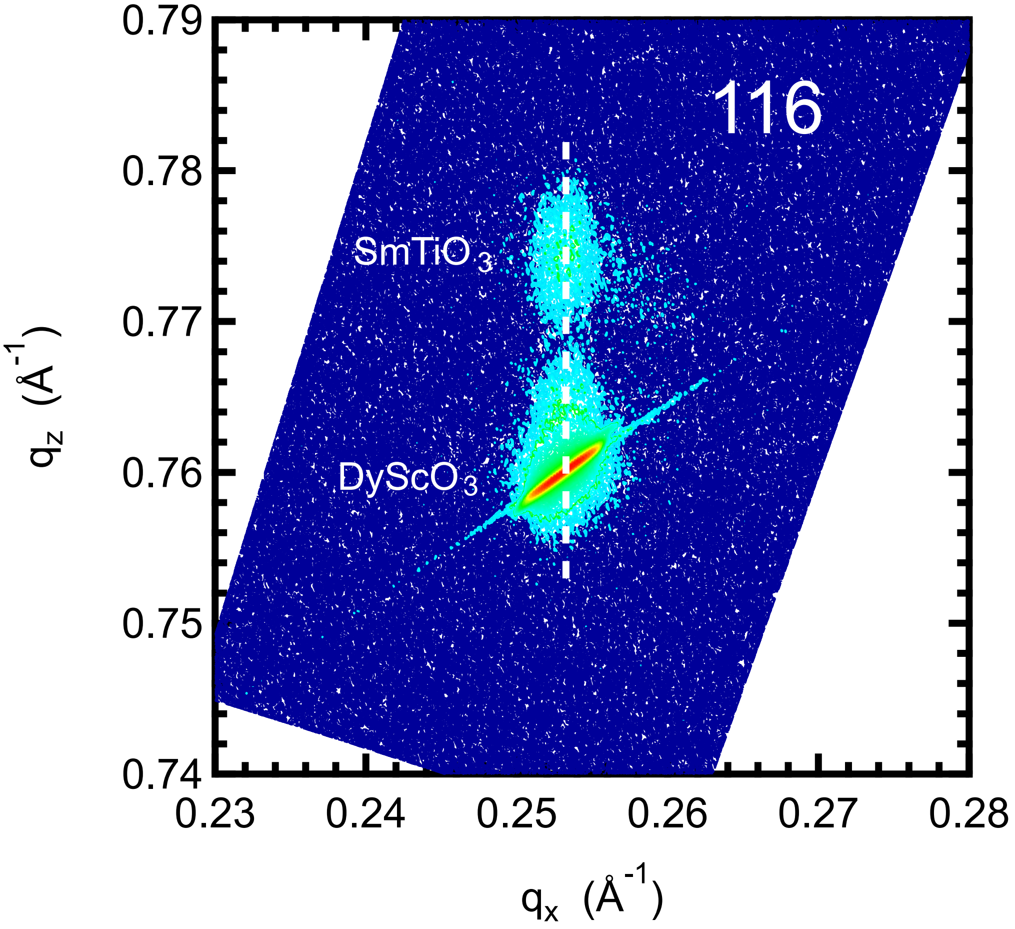

Figure S2. Reciprocal space map aligned to the orthorhombic 116 peak of a 10 nm $SmTiO_3$/10 SrO/10 nm $SmTiO_3$/$DyScO_3$ quantum well structure demonstrating that the $SmTiO_3$ layers are fully strained to the $DyScO_3$ substrate. The $DyScO_3$ substrate peak and $SmTiO_3$ film peak are labeled.
